# Chloride‐Reinforced Carbon Nanofiber Host as Effective Polysulfide Traps in Lithium–Sulfur Batteries

**DOI:** 10.1002/advs.201600175

**Published:** 2016-07-21

**Authors:** Lei Fan, Houlong L. Zhuang, Kaihang Zhang, Valentino R. Cooper, Qi Li, Yingying Lu

**Affiliations:** ^1^ State Key Laboratory of Chemical EngineeringInstitute of Pharmaceutical EngineeringCollege of Chemical and Biological EngineeringZhejiang UniversityHangzhou310027China; ^2^ Department of Mechanical and Aerospace EngineeringPrinceton UniversityNJ08544USA; ^3^ School of Materials Science and EngineeringZhejiang UniversityHangzhou310027China; ^4^ Materials Science and Technology DivisionOak Ridge National LaboratoryOak RidgeTN37831USA

**Keywords:** diffusion, high S loading, lithium–sulfur batteries, metal chlorides, surface adsorption

## Abstract

Lithium–sulfur (Li–S) battery is one of the most promising alternatives for the current state‐of‐the‐art lithium‐ion batteries due to its high theoretical energy density and low production cost from the use of sulfur. However, the commercialization of Li–S batteries has been so far limited to the cyclability and the retention of active sulfur materials. Using co‐electrospinning and physical vapor deposition procedures, we created a class of chloride–carbon nanofiber composites, and studied their effectiveness on polysulfides sequestration. By trapping sulfur reduction products in the modified cathode through both chemical and physical confinements, these chloride‐coated cathodes are shown to remarkably suppress the polysulfide dissolution and shuttling between lithium and sulfur electrodes. From adsorption experiments and theoretical calculations, it is shown that not only the sulfide‐adsorption effect but also the diffusivity in the vicinity of these chlorides materials plays an important role on the reversibility of sulfur‐based cathode upon repeated cycles. Balancing the adsorption and diffusion effects of these nonconductive materials could lead to the enhanced cycling performance of an Li–S cell. Electrochemical analyses over hundreds of cycles indicate that cells containing indium chloride‐modified carbon nanofiber outperform cells with other halogenated salts, delivering an average specific capacity of above 1200 mAh g^−1^ at 0.2 C.

High energy and safe electrochemical storage are critical components to meet the increasing demands for portable electronic devices as well as large‐scale energy storage systems.^1^, ^2^, ^3^ From smartphones, unmanned aircraft, to hybrid electrical vehicles, research into high energy lithium‐based batteries that provide higher specific capacity, larger power output, increased safety, and extended cycle life has received intensive consideration during the past decade.^4^, ^5^, ^6^, ^7^ The state‐of‐the‐art lithium‐ion battery (LIB) is almost approaching its theoretical performance limit (≈300 mAh g^−1^) due to the relatively low capacity of both graphite‐containing anode and transition metal‐based cathode materials. Among various alternatives, rechargeable batteries that use earth abundant elements and cost‐effective materials are considered to be promising candidates to meet these performance goals and potentially offer opportunities in large‐scale deployment. In particular, sulfur appeals to the battery community because of its high energy density on both volume (2.8 kWh L^−1^) and weight (2.5 kWh kg^−1^) basis.^7^, ^8^, ^9^, ^10^ Unlike the widespread LIBs that host lithium in its ionic state and generally yield fewer than one electron per metal atom, a lithium–sulfur battery achieves its high energy (theoretical capacity of 1675 mAh g^−1^) from the multi‐step electrochemical redox reactions by bonding to two Li ions non‐topotactically (S_8_ + 16Li ↔ 8Li_2_S) and offer up to two electrons per sulfur atom.

The well‐known challenges for Li–S batteries are associated with two main aspects: (1) poor ion and electron transport dynamics due to the uncontrolled dissolution of complex sulfur species and the insulating nature of sulfur (*σ* = 5 × 10^−30^ S cm^−1^)^11^ and its reduction compounds, sulfides (*σ* = 10^−13^ S cm^−1^);^12^, ^13^ (2) loss of active materials into electrolytes stemming from the shuttling of soluble lithium polysulfide (LiPS) intermediates. The poor conductivity limits the accessibility of active cathode materials and the insolubility nature in most organic solvents hinders the oxidation reactions. The sulfur electrode has low stability during the spontaneous conversion reduction of sulfur with lithium and can detach from the cathode host in the form of soluble S species. It increases the electrolyte viscosity and reduces the utilization of active materials, leading to rapid capacity decay and low Coulombic efficiency. Such process is understood to be more complicated because certain amount of soluble LiPS in electrolyte could provide an appreciable benefit regarding the thermodynamically sluggish reactions of Li_2_S and S.^13^, ^14^ It also facilitates the formation of a favorable passivation layer on the surface lithium anode which curbs further loss of active anode material from chemical reactions of lithium with electrolytes.^14^, ^15^ However, too many of LiPS species in the electrolyte would increase the viscosity, reduce the ionic conductivity, may stem the pores in the separator and more severely react with lithium in a cyclic mode without producing electrical energy. In a typical reduction process, solid sulfur generates high‐order LiPS between 2.4 and 2 V, then forms low‐order LiPS below 2 V, and ends up with insoluble Li_2_S_2_ and Li_2_S. During oxidation process, the insoluble Li_2_S_2_ and Li_2_S become sulfur via soluble sulfur complex. However, the high solubility of LiPS intermediates in commonly used electrolytes may also diffuse in the electrolyte and react chemically with the two electrodes to yield other S species. Such process is driven by the concentration gradient of LiPS which is termed shutting effect. It causes the specific capacity well below theoretical expectation and reduces the ability of electrical energy storage of an Li–S battery upon repeated cycles.

Various approaches for the confinement of these sulfur species in a cathode host have been intensively studied in order to overcome the problem caused by soluble LiPS. Cathode modification is a common method to effectively sequester LiPS by incorporating affinity additives. Graphene oxide,^16^, ^17^ metal oxides/sulfides,^18^, ^19^, ^20^, ^21^ polymers,^9^, ^22^, ^23^ and bifunctional binders^24^, ^25^ have been widely studied to constrain active cathode materials by the high binding energy between sulfur species and O,N‐containing functional groups. These studies have indicated that stronger interactions between the polar group from the conductive materials (e.g., oxides and sulfides) and the S species enable better confinement of Li_2_S*_x_* and enhance the cycling performance of an Li–S cell. It is also suggested that conducting substrate could facilitate the electron transfer along the cathode host and favor the sluggish redox reactions of the insulating sulfur species. However, from a different mechanism that reported recently,^26^ non‐conductive metal oxides on a carbon substrate also have remarkable ability of trapping LiPS and promote the electrochemical properties. Due to the nonconductive nature of these oxides, it works together with the conductive carbon matrix to improve the conductivity of the sulfur hosts. Both the adsorption of Li_2_S*_x_* on the nonconductive traps and the diffusion from the nonconductive traps to the conductive substrate should be considered. There is no direct electron transfer route between the trapped Li_2_S*_x_* and the nonconductive materials, thus these S species should be transferred to the conductive carbon substrate for further electrochemical reactions. Too strong binding between nonconductive materials and Li_2_S*_x_* could indeed impair the proper function of Li–S batteries because trapping Li_2_S*_x_* too tight on insulating substrates would hinder the electron transfer and deactivate S materials. Therefore, intermediate binding between nonconductive materials and the S species is favorable.

In this work, we report a facile synthesis procedure and electrochemical properties of a family of nonconductive chloride‐reinforced carbon nanofiber/sulfur composites that incorporate spatial control of charge/discharge products in the cathode in order to minimize the loss of soluble S species. Conductive carbon nanofibers have recently attracted great interest as effective polysulfide traps due to the opportunities they provide for tunable surface area, structure, and chemistry. When attached to a common separator, the micropores in the interspace of the 3D carbon nanofiber can effectively hinder the polysulfide diffusion and migration.^27^, ^28^ Hollow structures can also be created in each nanofiber in order to increase the space for hosting polysulfides.^8^, ^29^, ^30^, ^31^ More interestingly, binder‐free solid carbon nanofibers with or without surface decoration have been reported as free‐standing sulfur cathode.^10^, ^32^, ^33^ The empty space among nanofibers is sufficient to host sulfur and accommodates the volume change during the redox reactions. The electrode surfaces are designed to have proper affinity with S species in order to control the deposition of S species uniformly and avoid large aggregates of the deposits thus increasing the utilization of active materials. Besides acting as a physical barrier, an ideal sulfur host should also possess strong chemical interaction with polar Li_2_S and LiPS species for further confinement of these species within the cathode structure during cycling. It should also promote the redox kinetics of polysulfides on the surface of the electrode. While previous studies have focused on the capture of polysulfides via metal oxides or sulfides, the effectiveness of halogenated salts on polysulfides confinement has been rarely reported. Based on systematic simulations and adsorption experiments of interactions between halogenated salts and S compounds, we proved their abilities of confining polysulfides. We then selected three chlorides out of more than 20 halogenated salts with various interactions of S compounds. These chlorides are designed to increase the polarity of a common conductive carbon‐based host which has relatively weak binding ability because of their nonpolar nature. On the other hand, the conductive carbon helps the electron transfer between Li_2_S*_x_* and the cathode host. The nonconductive chlorides and conductive carbon nanofibers work together to promote the cycling performance of an Li–S cell by balancing the adsorption and diffusion processes. Indium chloride‐modified carbon nanofiber with moderate interaction with Li_2_S and LiPS outperforms the other chloride modifications. It delivers a high discharge capacity of 1217 mAh g^−1^ at 0.2 C and stable long‐term cyclability at both intermediate and high S loadings.

As shown in Figure S1 (Supporting Information), more than 20 halogenated salts were considered as absorbents for sulfur materials and their binding abilities were evaluated by the color changes of Li_2_S_8_ solutions. Three chloride materials, CaCl_2_, InCl_3_, and MgCl_2_ were selected based on their rapid color change and could be effective materials for sequestering LiPS. In the Supporting Information, we also showed the simulation results of two other candidate chloride materials, MnCl_2_ and CrCl_3_. However, we here focus on the discussions on the other three chloride materials without any loss of generality. **Figure** **1** shows the schematic representations of the optimized atomic positions of Li–S species absorbed on CaCl_2_ (a), InCl_3_ (b), and MgCl_2_ (c), respectively. Density functional theory (DFT) calculations were performed via the Vienna Ab Initio package (VASP) in the generalized gradient approximation (GGA) with the Perdew–Burke–Ernzerhof (PBE) parameterization (see details in the Supporting Information). The effectiveness of the entrapment of S species in the cathode was determined by the binding energy between the Li–S species and the three chlorides, which is defined as, *E*_b_ = *E*_slab_ + *E*_Li–S_ − *E*_slab+Li–S_, where *E*_slab_ refers to the energy of the surface slab in the absence of the Li–S species, *E*_Li–S_ denotes the energy of the Li–S species, and *E*_slab+Li‐S_ is the energy of the Li–S species adsorbed on the surface slab of a chloride material. Previous results indicate that entities that bind strongly to Li_2_S also have strong interactions with other S species owing to their similar chemical bonding nature.^24^, ^34^ We corrected the *E*_b_ by applying the vdW‐DF2 (van der Waals density functional). Including the vdW corrections, the interactions with Li_2_S increase, following the order: CaCl_2_ > InCl_3_ > MgCl_2_ (summarized in Table S1, Supporting Information). Physical immobilization of sulfur and its reduction products plays an important role at the beginning of lithiation stage and could contribute more than 25% of the total interactions, especially for weak anchoring molecules.^34^ According to the simulation results, the interactions between chlorides and Li–S are much stronger than a single layer of carbon‐based graphene (0.29 eV). The rapid increase in binding energy via chloride dopants can be explained by their polar nature. It also suggests that the dual effect of physical confinement and chemical binding of the chloride compounds enables strong entrapment of Li_2_S and LiPS within the cathode during cycling. The binding energy between Li–S and a single layer of CaCl_2_ was calculated to be 3.37 eV, which is more than twice of the other two species. However, too strong chemical binding, typically *E*_b_ > 2.0 eV could essentially reduce the amount of active sulfur content and impair the cyclability of an Li–S cell. When the binding between Li and the sorbent is strong enough to compete the binding between Li and S, Li ion could prefer to stay on the cathode instead of forming Li–S bond. It could cause the separation of the two elements and deteriorates the integrity of Li_2_S and LiPS.^34^ Moreover, due to the poor conductivity of the chlorides, the absorbed LiPS should diffuse to the surface of carbon nanofibers for proper electrochemical reaction to proceed. Therefore, intermediate interaction between sulfur species and chloride materials might be a reasonable choice.

**Figure 1:**
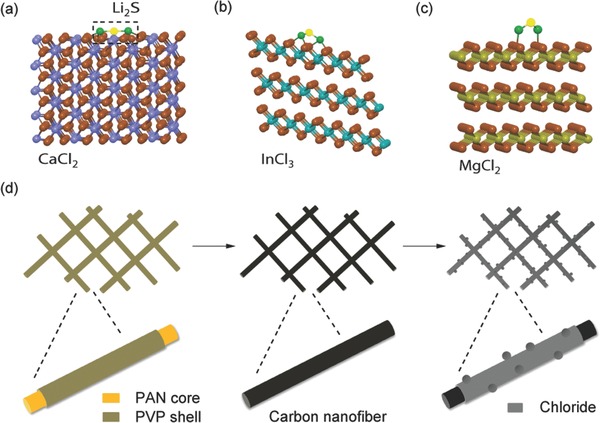
d) Schematic representation of Li_2_S entrapment and the synthesis procedure of chloride‐reinforced carbon nanofibers. It illustrates the optimized atomic positions of Li–S species adsorbed on a) CaCl_2_, b) InCl_3_, and c) MgCl_2_, respectively. PVP is removed from the hybrid fiber through thermal treatment.

Figure 1d exhibits the synthesis procedure of chloride‐reinforced carbon nanofiber. The free‐standing carbon nanofiber was decorated with chlorides and utilized as a binder‐free sulfur host. Polyacrylonitrile (PAN)/polypyrrolidone (PVP) hybrid nanofiber was fabricated via co‐electrospinning method. It formed a core–shell structure with PAN as the core and PVP as the shell by a phase‐separated solution. The resultant nanofiber was calcined in a box furnace under air atmosphere at 300 °C for 4 h, to yield oxidized PAN nanofibers due to the thermal decomposition of PVP. PVP was introduced in the electrospinning step in order to increase the homogeneity of the fiber and increase the amount of O,N‐containing groups on the fiber.^35^ The oxidized PAN nanofiber was then carbonized under Ar protection at 850 °C for 2 h, to form conductive carbon nanofibers. Chloride materials were coated on the surface of carbon nanofiber via physical vapor deposition (PVD). Their affinity on the carbon nanofiber was demonstrated by the absence of In and Cl signals in the electrolyte and on the lithium surface, which will be discussed in the sections below.

The morphologies of the as‐synthesized chloride‐reinforced CNF were analyzed by scanning electron microscope (SEM) and transmission electron microscopy (TEM). **Figure** **2**a–c shows typical SEM images of InCl_3_‐coated CNF at different magnifications (also see Figure S2, Supporting Information). It indicates that the material exists in a non‐woven nanofiber structure with average fiber diameter between 200 and 300 nm. In contrast to the smooth surface of bare carbon nanofiber (Figure S3, Supporting Information), many small nanoparticles with diameter smaller than 100 nm are assembled to the surface of InCl_3_‐CNF. The corresponding elemental scanning of indium (Figure S4, Supporting Information) on carbon nanofiber also reveals the uniform distribution of InCl_3_ on CNF. TEM images in Figure 2d–f are consistent with the results obtained from SEM analysis. InCl_3_ particles attached on carbon nanofibers are darker in color compared with the conductive carbon, suggesting the existence of heavy metal. High‐resolution TEM images in Figure 2f show that the nanoparticles are crystalline while the carbon substrate is amorphous. The inset of Figure 2f is the selected area electron diffraction (SAED) pattern of InCl_3_ on carbon nanofiber, suggesting the crystalline structure of the InCl_3_‐CNF composite. Energy dispersive X‐ray spectroscopy (EDX) spectrum (Figure 2g) and scanning transmission electron microscopy (STEM) images (Figure 2h–j) were obtained to illustrate the distribution of InCl_3_ on CNF. InCl_3_ coating not only exists as crystalline InCl_3_ nanoparticles but also smoothly embraces the carbon nanofiber. Consistent with the results, line scan profiles of the cross section of one typical InCl_3_ coated CNF in Figure S5 (Supporting Information) also suggest that the carbon nanofiber is decorated by a thin layer of InCl_3_ and small InCl_3_ nanoparticles. Similar synthesis method was applied for the preparation of CaCl_2_ and MgCl_2_‐coated CNF and their morphologies are provided in Figure S6 (Supporting Information).

**Figure 2:**
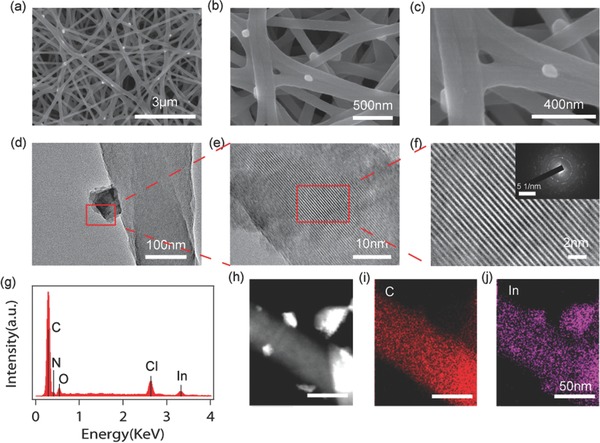
Morphologies and element identifications of the as‐synthesized indium chloride‐reinforced CNF. a–c) SEM and d–f) TEM images of InCl_3_‐coated CNF with different magnifications. The inset of (f) is the SAED pattern of InCl_3_ on carbon nanofiber. g) EDX spectrum under SEM mode. h) STEM image and EDX maps of i) carbon and j) indium distributions under TEM mode. The scale bars of (h)–(j) are the same.

The effectiveness of the different chloride‐reinforced CNF on polysulfide confinement is studied by cycling Li/S cells under galvanostatic condition. The electrochemical measurements were carried out in a coin‐cell configuration with metallic lithium foil as the anode. Sulfur was added to the free‐standing carbon nanofiber in the form of Li_2_S_8_. Specifically, pre‐determined amount of Li_2_S and S was dissolved in 1,3‐dioxolane (DOL)/1,2‐dimethoxyethane (DME) (1:1 in volume) solution under moderate heating at 60 °C, to yield 5 m Li_2_S_8_ solution. 12.5 μL of 5 m Li_2_S_8_ solution was dropped on the CNF substrate to make the sulfur loading as 2 mg cm^−2^. After evaporation of the organic solvents, solid sulfur content was remained in the CNF host. 40 μL of 1 m LiTFSI in DOL/DME with 1 wt% LiNO_3_ as additive was then added as electrolyte to evaluate the electrode. **Figure** **3**a displays the cycling performance of Li–S batteries with bare CNF, and with InCl_3_, CaCl_2_, or MgCl_2_‐coated CNF at 0.2 C. The corresponding charge/discharge profiles at initial, 50th, 100th, and 200th cycles of cells with bare CNF, and with InCl_3_, CaCl_2_, or MgCl_2_‐coated CNF are provided in Figure S7 (Supporting Information). It is clear from the figure that the enhancement in specific capacity is achieved compared to cathode without chloride additives, following the order: InCl_3_‐CNF ∼ CaCl_2_‐CNF > MgCl_2_‐CNF > bare CNF. While the binding energy of CaCl_2_ is much higher than InCl_3_, the InCl_3_‐based cathode shows similar capacity to CaCl_2_‐based cathode but higher Coulombic efficiency than CaCl_2_‐based cathode. These results could associate with the band gaps, adsorption, and diffusion factors with the two materials. Cells with InCl_3_‐coated CNF deliver a capacity of 1217 mAh g^−1^ over 200 cycles. Cells using MgCl_2_‐CNF have stronger interactions with lithium polysulfides compared with bare CNF and consequently provide enhanced cycling performance over bare CNF. Consistent with the discussions reported in the previous section, it suggests that the cycling performance is not only associated with the interactions of chloride with S species, but also closely related to the diffusion of the trapped S species to the conductive carbon nanofibers.^26^ Too strong binding of polysulfides with nonconductive metal chlorides may ultimately impair the electrochemical properties of an Li–S cell because electrochemical redox reactions can hardly happen on the surface of nonconductive materials. And too weak interactions are also not suggested because S species could diffuse away from the cathode host and lead to severe shuttling problem. Based on the excellent cycling behavior of cells containing InCl_3_, we studied it in details. Figure 3b shows the first discharge profiles of cells with InCl_3_‐coated CNF and bare CNF. Bare CNF electrode renders a low capacity of only 661 mAh g^−1^ while the InCl_3_‐coated CNF electrode shows 1385 mAh g^−1^ in capacity. Cells containing bare CNF and InCl_3_‐coated CNF deliver similar specific capacity between 2.3 and 2.1 V, suggesting that the conversion from Li_2_S_8_ to Li_2_S_4_ has similar efficiency. It also can be seen that much of the capacity enhancement is from the second discharge plateau at around 2.1 V, corresponding to the phase change from liquid Li_2_S_4_ to solid Li_2_S_2_ or Li_2_S. The confinement of LiPS plays an important role on the conversion process from soluble LiPS to insoluble S species, and the capacity enhancement from the second discharge plateau is more obvious. The results indicate that the binding between InCl_3_ and small sulfur species is significant and the balance between LiPS adsorption on the metal chlorides and its subsequent diffusion to the carbon substrate is more favorable at around 2.1 V, which suppresses the shuttling problem and favors the electrochemical reactions.

**Figure 3:**
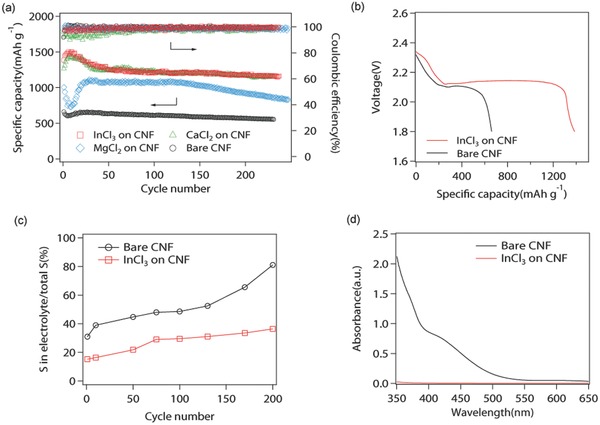
Cycling behavior of Li–S batteries with bare CNF, and with InCl_3_, CaCl_2_, or MgCl_2_‐coated CNF at intermediate sulfur loading of 2 mg cm^−2^. Cycle life was carried out at a constant 0.2 C rate of discharge and charge. a) Specific capacity and Coulombic efficiency as a function of cycle number. b) Initial discharge/charge voltage profiles of CNF with and without InCl_3_. c) Sulfur content in electrolytes at different cycles. d) UV–vis spectra of the electrolyte solutions obtained from cells with and without InCl_3_, after cell resting for 7 d.

The interface properties and the conductivities of the modified cathode were elucidated by impedance spectra of Li–S cells before cycling and after 200 cycles (Figure S8, Supporting Information). The measurements were performed with 10 mV perturbation amplitude in the range from 200 kHz to 0.1 Hz. The impedance spectra were recorded at each state allowing at least 12 h for equilibration after each charge/discharge step. At initial state, the Nyquist plots consist of an out‐of‐shape semicircle alike in high and intermediate‐frequency ranges and an inclined straight line in the lower frequency range. The semicircle is associated with the SEI layer formed on the Li anode and the straight line is related to the diffusion of ions. An equivalent circuit model is provided in Figure S8c (Supporting Information). Cells using InCl_3_‐coated CNF show a smaller semicircle in the high and moderate‐frequency region compared to cells with bare CNF, which indicates lower charge‐transfer resistance in the former case. After 200 cycles, semicircles with compressed shape appear in both high and intermediate‐frequency range, which is associated with passivating film and charge‐transfer resistance, respectively. It can be found that in both cases, the semicircles that appear in the high‐frequency range show no obvious change upon repeated cycles, which indicates that the passivation film is stable during latter charge/discharge cycles. In the case of InCl_3_‐coated CNF, it shows a smaller semicircle in the moderate‐frequency range, revealing that charge‐transfer resistance remains low upon repeated cycles.

Quantitative experiments were carried out to evaluate the loss of polysulfide to the electrolyte during the recharge process. The trace of S species in the electrolytes at different cycles was monitored by inductively coupled plasma (ICP) and UV–vis spectra, as shown in Figure 3c,d. It exhibits that sulfur concentrations in the electrolytes obtained from cells with InCl_3_‐coated CNF remain low while sulfur element in the electrolytes increases upon repeated cycles and can be clearly detected in the cells with bare CNF. UV–vis spectra, harvested from cells after resting for 7 d, also show strong absorption signals of polysulfides around 400–450 nm for the case of cells containing bare CNF, but almost negligible peaks for cells using InCl_3_‐coated CNF. It confirms that the access of polysulfide to the electrolytes is limited by using InCl_3_‐coated CNF and InCl_3_ could be an effective absorbent for confining LiPS in the cathode.

The morphologies of S species that deposit on the carbon nanofibers after 200 cycles were studied by SEM (**Figure** **4**a,b). The electrodes were obtained after discharge and washed by DOL/DME solvent to remove the soluble S species. Large particles in micrometer size can be found on bare CNF electrode while no obvious particles appear on the InCl_3_‐modified CNF. Morphologies with different magnifications can be found in Figure S9 (Supporting Information). Figure S9c (Supporting Information) shows that the diameter of InCl_3_–C nanofibers increases and InCl_3_–C nanofibers were smoothly coated with a layer of S species. It can be explained by the nonpolar nature of carbon substrate. Thus, sulfur reduction products tend to aggregate and form large deposits. Once modified by InCl_3_, the carbon nanofibers have more favorable sites for Li*_x_*S deposition. The chemistry of the deposits on the two nanofibers was identified by EDX, as shown in Figure S10 (Supporting Information). The large particles that appear on the SEM image (Figure S10d, Supporting Information) show more sulfur signal on the EDX map, as indicated in white circles (Figure S10f, Supporting Information). The sulfur elemental distribution is more homogenous on InCl_3_–C composite than that on bare carbon substrate. Consistent with the finding in the previous section, InCl_3_ modification increases the interactions between S species and the host and creates more sites to confine the polysulfides on the surface of carbon nanofibers. It promotes the subsequent redox reactions by the adsorption‐diffusion effect and increases the active material utilization.

**Figure 4:**
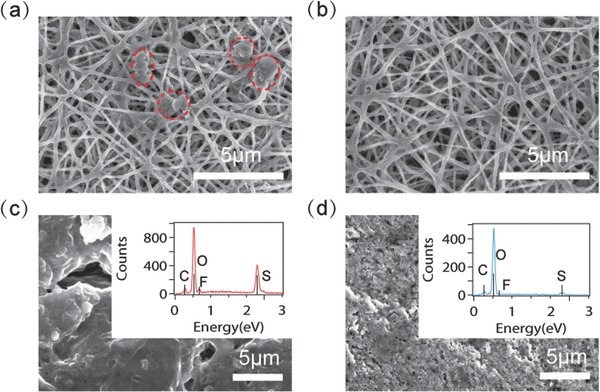
Post‐mortem SEM analyses of electrodes obtained after 200 cycles. Morphologies of S species formed on b) bare CNF and b) CNF with InCl_3_. Morphologies and EDX spectra (insets) of lithium harvested from cells with c) bare CNF and d) InCl_3_–C nanofiber.

As discussed earlier, the InCl_3_ on CNF reduces the polysulfide dissolution and Li–S cells that using InCl_3_‐CNF exhibit high specific capacity and Coulombic efficiency. To further confirm these effects are generated by the interactions of S species with InCl_3_ and favorable S utilization triggered by the diffusion of S species from InCl_3_ sites to C substrate, SEM and EDX analyses were also carried out for the counter lithium electrode after 200 cycles, as displayed in Figure 4c,d. The surface of the lithium foil obtained from the cell using InCl_3_–C electrode is porous and relatively uniform compared with that from the cells using bare CNF. It indicates that the SEI layer formed on the lithium anode in the cells using InCl_3_‐CNF can protect lithium and suppress lithium dendrite formation, enabling stable cycling performance. The inset EDX spectrum shows very weak signal of sulfur, suggesting that the shuttling problem can be effectively suppressed by using InCl_3_–C nanofiber cathode host. The solubility of polysulfides in electrolytes is not fully eliminated by InCl_3_ capture due to its moderate adsorption ability; indeed, we intend to allow some soluble polysulfide in order to form a protective film on the lithium surface and facilitate reversible charge/discharge because the solid–solid reaction between S_8_ and Li_2_S*_m_* is thermodynamically slow at room temperature. Moreover, the intermediate adsorption with Li_2_S*_x_* on the nonconductive chlorides enables better accessibility of conductive carbon with Li_2_S*_x_*, improving the subsequent electrochemical reactions. It is also important to realize that too strong binding of S species on the sorbents may reduce the interaction between Li and S, and eventually reduce the amount of active material. In Figure 4d, chlorine signal is not found around 2.6 eV, meaning no observations of detachment of InCl_3_ from carbon nanofiber upon repeated cycles. And the effectiveness of InCl_3_ is on the carbon substrate, neither in the electrolyte nor on the lithium anode.

The long‐term cycling stability and rate capability of cells using InCl_3_‐reinforced carbon nanofiber were further examined by galvanostatic cycling measurement at room temperature. **Figure** **5**a reports the cycling performance of Li–S with InCl_3_–C nanofiber at high sulfur loading, 4 mg cm^−2^ (3 mg of the nanofiber host with 4 mg sulfur doped in the form of Li_2_S_8_) over 650 cycles at 0.2 C. Cells using modified electrode can cycle stably and deliver high specific capacity of 1088 mAh g^−1^ with a small capacity degradation of 0.019% cycle^−1^ from 5th to 650th cycle. Even after 650 cycles, the capacity still remains as high as 891 mAh g^−1^. It can be explained by its exceptional ability of hosting S species and avoiding the structure failure generated by volume change during cycling. InCl_3_ increases the ability of trapping S species in the cathode thus reducing the loss of S species in the electrolyte while the 3D structure of carbon nanofiber could withstand the large volume change of the cathode during cycling. The cyclabilities of cells using bare CNF and InCl_3_‐CNF without 1 wt% LiNO_3_ additives were also examined at 0.2 C, as shown in Figure S11 (Supporting Information). The capacity of cells with bare carbon nanofiber decays very fast and can only deliver 319 mAh g^−1^ after 50 cycles. Cells that utilize modified electrode exhibit stable cycling after ten cycles with capacity maintained at 951 mAh g^−1^, which is more than twice of the former one. Figure S11b (Supporting Information) shows that without LiNO_3_, the InCl_3_–C hybrid material improves the first cycle Coulombic efficiency by 41.6%, which further proves its effectiveness of suppressing polysulfide shutting. Figure 5b,c exhibits the rate performance and the corresponding voltage profiles at various C rate for cells that utilize InCl_3_–C nanofibers. 20 cycles at each fixed current densities, from 0.2 to 1.6 C, were investigated. When the C rate was increased from 0.2, 0.4, 0.8 to 1.6 C, the cells provide high stabilized capacities of 1203, 1095, 1044, and 981 mAh g^−1^ at 0.2, 0.4, 0.8, and 1.6 C, respectively. After cycling at 1.6 C, the original capacity can be successfully recovered to 1178 mAh g^−1^ when switched back to 0.2 C. The charge and discharge profiles show similar characteristics at all current densities studied. Figure 5d shows that the overpotentials increase slightly with the rapid increase of current density, suggesting reasonable conductivity and charge‐transfer kinetics of the cathode materials even at high C rates.

**Figure 5:**
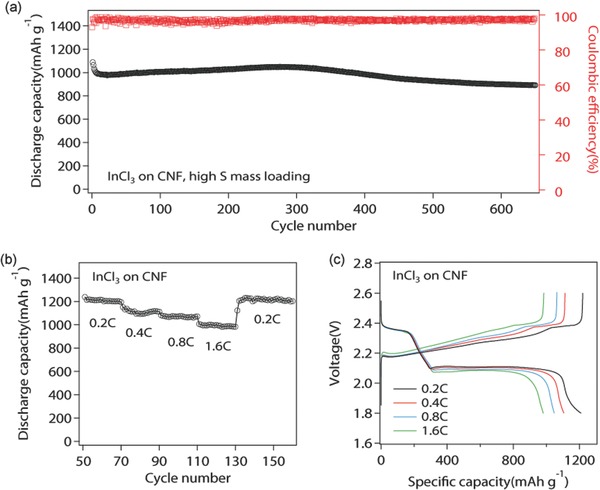
a) Long‐term cycling performance of Li–S batteries with InCl_3_–C nanofiber with high sulfur loading, 4 mg cm^−2^ over 650 cycles at 0.2 C. b) Rate capability and c) the corresponding voltage profiles of cells containing InCl_3_ at moderate sulfur loading, 2 mg cm^−2^. The specific capacities were calculated based on sulfur.

In summary, we created a family of chloride‐reinforced carbon substrates for sequestering S species in Li–S batteries. It is known to be the first time to demonstrate the ability of nonconductive halogenated salts for the suppression of polysulfides dissolution in electrolytes. We calculated the binding energies between chlorides and Li–S via ab initio simulations, and corrected the results by incorporating vdW interactions. The effectiveness of polysulfide confinement was examined by galvanostatic cycling. Cells that use indium chloride modified electrodes outperform cells with other chloride modifications and show high average specific capacity of 1217 mAh g^−1^ with moderate sulfur loading 2 mg cm^−1^ at 0.2 C. It indicates that intermediate interaction between InCl_3_–C nanofiber and S species might be more desirable for polysulfides entrapment by balancing the adsorption and diffusion effects. Allowing small amount of polysulfide dissolution may also favor the SEI stabilization as well as promote the redox reactions of solid‐state S species. More aggressive measurements were also carried out to evaluate its cyclability and rate capability. When loaded with high sulfur content, the cells are able to cycle stably over 650 cycles with only 0.019% capacity decay per cycle from 5th to 650th cycles.

## Experimental Section

*Materials Synthesis*: 0.5 g PAN (*M*_w_ = 150 000) and 0.5 g PVP (*M*_w_ = 1 300 000) were dissolved in 10 mL dimethylformamide (DMF) with rigorous stirring for 12 h at 60 °C. The resultant solution was continuously deposited on a conductive carbon paper for 4 h at a rate of 0.3 mL h^−1^ (LSP01‐1A, LongerPump) using a conventional electrospinning setup. The distance of the air gap is 18 cm and the static electric voltage is set at 15 kV. The polymer nanofibers covered carbon paper was then transferred to a box furnace (Lindberg Blue M, Thermo Scientific) and oxidized at a constant temperature 300 °C for 4 h. After the oxidation step, PVP shell was removed from the polymer nanofiber and PAN core was oxidized and formed a free‐standing film. The product was then carbonized in a tube furnace (Lindberg Blue M, Thermo Scientific) under Ar protection at 850 °C for 2 h, to yield a carbon nanofiber film. The as‐prepared carbon nanofibers were treated by 3 m KOH ethanol solution to increase the surface area, from 5 to 222 m^2^ g^−1^. To coat chlorides on the carbon nanofiber, 0.06 g InCl_3_ powder was placed in the center of a tube furnace and deposited on the carbon nanofibers film (0.2 g) via PVD under continuous Ar flow. The deposition of MgCl_2_ or CaCl_2_ on carbon nanofibers was prepared by the same method.

*Characterization*: The morphologies and elemental identifications of carbon nanofibers were analyzed by SEM (SU‐8010, Hitachi) and TEM (200 kV‐2100F, JEOL). For post‐mortem SEM studies, cells were stopped at the discharge step and disassembled in an Ar‐filled glovebox (Mikrouna). And the lithium foil and carbon nanofiber electrode were harvested, washed with DOL/DME solvent, and dried in an anti‐chamber before the analysis. The sulfur concentrations in the electrolytes were measured as a function of cycle number via ICP‐MS (NexION300X, ParkinElmer). UV spectrometer (UV‐1800, Shimadzu Corporation) was used to analyze the polysulfide content in the electrolyte after 200 cycles. The surface area of the KOH‐etched carbon nanofiber was calculated by BET (3FLEX, Micromeritics).

*Electrochemical Measurements*: The electrolyte used was a solution of 1 m bis(trifluoromethane)sulfonimide lithium salt (LiTFSI) in DOL/DME (1:1 in volume) containing 1 wt% LiNO_3_ as additive. Sulfur in the cathode was added in the form of Li_2_S_8_. 12.5 or 25 μL of 5 m Li_2_S_8_ was dropped onto the carbon substrate and dried under Ar protection. The thickness of the electrode is around 90 μm. 2023 type coin‐cells were assembled under Ar protection using lithium metal (Alfa Aesar, 0.75 mm) as the anode, a porous film (Celgard 2500) as the separator, Li_2_S_8_ on modified carbon nanofiber as the cathode, and 40 μL electrolyte for each cell. Impedance measurements were performed using Solartron Analytical (1470E) in the frequency range of 200 kHz to 0.1 Hz. Galvanostatic cycling in the voltage range of 1.8–2.6 V (vs Li^+^/Li) was carried out using eight‐channel Neware battery tester (CT‐3008) in a temperature chamber (BLC‐300).

## Supporting information

As a service to our authors and readers, this journal provides supporting information supplied by the authors. Such materials are peer reviewed and may be re‐organized for online delivery, but are not copy‐edited or typeset. Technical support issues arising from supporting information (other than missing files) should be addressed to the authors.

Supplementary
