# Supplementary material for: Chloride‐Reinforced Carbon Nanofiber Host as Effective Polysulfide Traps in Lithium–Sulfur Batteries
Source: Adv Sci (Weinh). 2016 Jul 21;3(12):1600175. doi: 10.1002/advs.201600175 (PMC5157171; doi:10.1002/advs.201600175)
Supplement: Supplementary file 1 — Supplementary [file ADVS-3-0-s001.pdf]

Copyright WILEY-VCH Verlag GmbH & Co. KGaA, 69469 Weinheim, Germany, 2016.

ADVANCED SCIENCE
Open Access

# Supporting Information

for *Adv. Sci.,* DOI: 10.1002/advs.201600175

# Chloride-Reinforced Carbon Nanofiber Host as Effective Polysulfide Traps in Lithium–Sulfur Batteries

*Lei Fan, Houlong L. Zhuang, Kaihang Zhang, Valentino R. Cooper, Qi Li, and Yingying Lu**

**Supporting Information**

Theoretical methods

We perform the density-functional theory calculations using the Vienna *Ab Initio* package (VASP)[1] in the generalized gradient approximation (GGA) with the Perdew-Burke-Ernzerhof (PBE) parameterization.[2-3] The GGA version of projector augmented wave (PAW) pseudopotentials is used where the $1s^2 2s^1$ electrons of Li, $2s^2 2p^6 3s^2$ electrons of Mg, $3s^2 3p^4$ electrons of S, $3s^2 3p^5$ electrons of Cl, $3s^2 3p^6 4s^2$ electrons of Ca, $3s^2 3p^6 3d^5 4s^1$ electrons of Cr, $3s^2 3p^6 3d^5 4s^2$ electrons of Mn, and $4d^{10} 5s^2 5p^1$ electrons of In are treated as valence states.[4] We use a cutoff energy of 500 eV for the plane wave basis sets to expand the wave functions. To simulate the chloride surfaces, we use nine atomic layers in the supercells. In addition, we fix the in-plane lattice constants of the supercells to 6 × 6, 4 × 4, 6 × 6, 4 × 4, and 3 × 2 of the calculated bulk lattice constants of $MgCl_2$, $CaCl_2$, $MnCl_2$, $CrCl_3$, and $InCl_3$. This result in five surface slabs consisting of 324, 432, 324, 384, and 288 atoms, respectively. A vacuum spacing of 20 Å is used to separate the image interactions between the simulation supercells. For the bulk calculations, we use Γ-point centered 8 × 8 × 2, 4 × 4 × 6, 8 × 8 × 2, 5 × 5 × 2, 4 × 2 × 4 *k*-point meshes for $MgCl_2$, $CaCl_2$, $MnCl_2$, $CrCl_3$, and $InCl_3$, respectively. However, we employ only the Γ-point for the surface calculations. For the calculations on $MnCl_2$ and $CrCl_3$, we also consider spin-polarizations which lead to ferromagnetic ground states of these two materials. We adopt effective $U$ parameters of 4.0 and 2.63 eV to deal with the $d$ orbitals of Cr and Mn atoms,[5,6] respectively. Atomic positions in the surface slabs are completely optimized until the Hellman-Feynman forces are converged to 0.03 eV/Å. Similar to previous studies, the binding energy $E_b$ between the Li-S species and the three chlorides are defined as,[7]

$$E_b = E_{slab} + E_{Li\text{-}S} - E_{slab+Li\text{-}S},$$

where $E_{slab}$ is the energy of the surface slab in the absence of the Li-S species, $E_{Li\text{-}S}$ is the energy of the Li-S species located in a vacuum box, and $E_{slab+Li\text{-}S}$ is the energy of the Li-S species adsorbed on a surface slab of chloride. Furthermore, we correct the $E_b$ by applying the vdW-DF2 van der Waals density functional,[8,9] which has also been used in a previous report on the interactions between the Li-S species and various two-dimensional layered materials.[10]

Supporting Figures

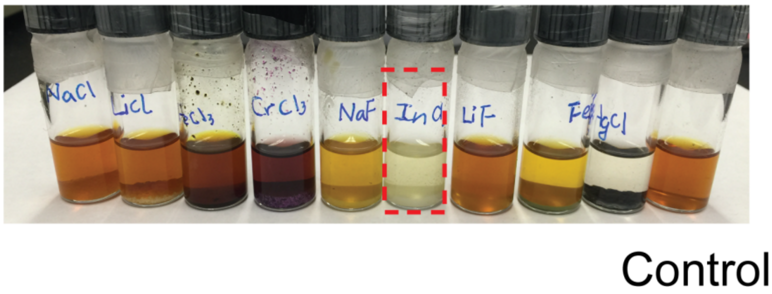


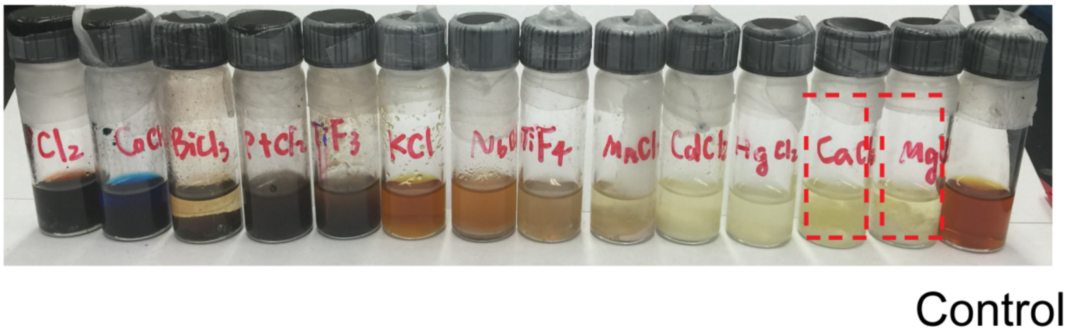


Figure S1. Color changes of $Li_2S_8$ solutions after doping different halogenated salts. The materials from the upper left to bottom right is NaCl, LiCl, $FeCl_3$, $CrCl_3$, NaF, $InCl_3$, LiF, $FeF_3$, AgCl, control sample, $CuCl_2$, $CoCl_2$, $BiCl_3$, $PtCl_2$, $TiF_3$, KCl, $NbCl_3$, $TiF_4$, $MnCl_2$, $CdCl_2$, $HgCl_2$, $CaCl_2$, $MgCl_2$, and control sample, respectively.

Table S1. Binding energies of Li-S with the three chlorides calculated without and with the vdW corrections.

| Chloride | Binding energy without vdW(eV) | Binding energy with vdW(eV) |
|---|---|---|
| $MgCl_2$ | 0.237 | 0.394 |

| | | |
|---|---|---|
| $CaCl_2$ | 3.081 | 3.370 |
| $MnCl_2$ | 0.584 | 0.733 |
| $CrCl_3$ | 1.298 | 1.842 |
| $InCl_3$ | 0.865 | 1.169 |

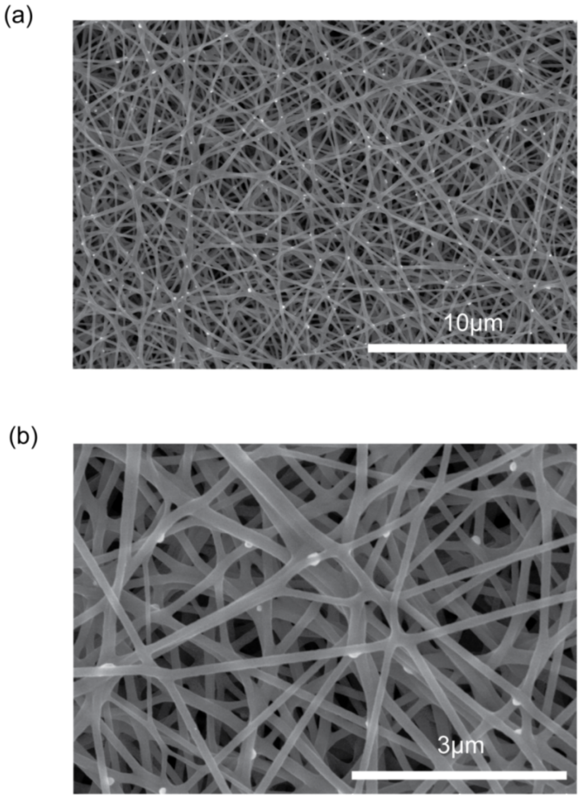


Figure S2. SEM images of $InCl_3$-coated carbon nanofiber.

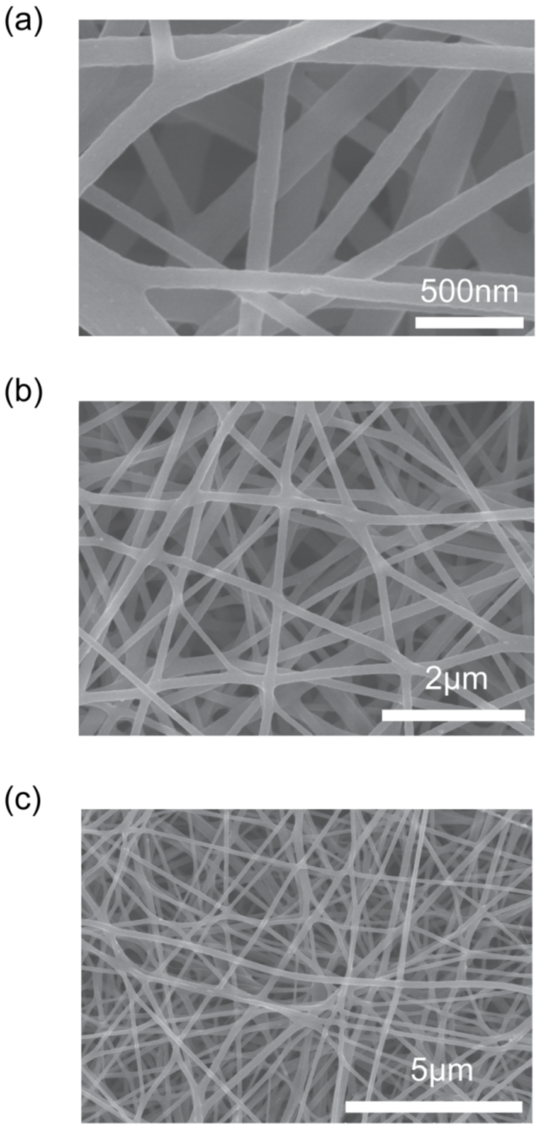


Figure S3. SEM images with various magnifications showing the morphologies of carbon nanofibers.

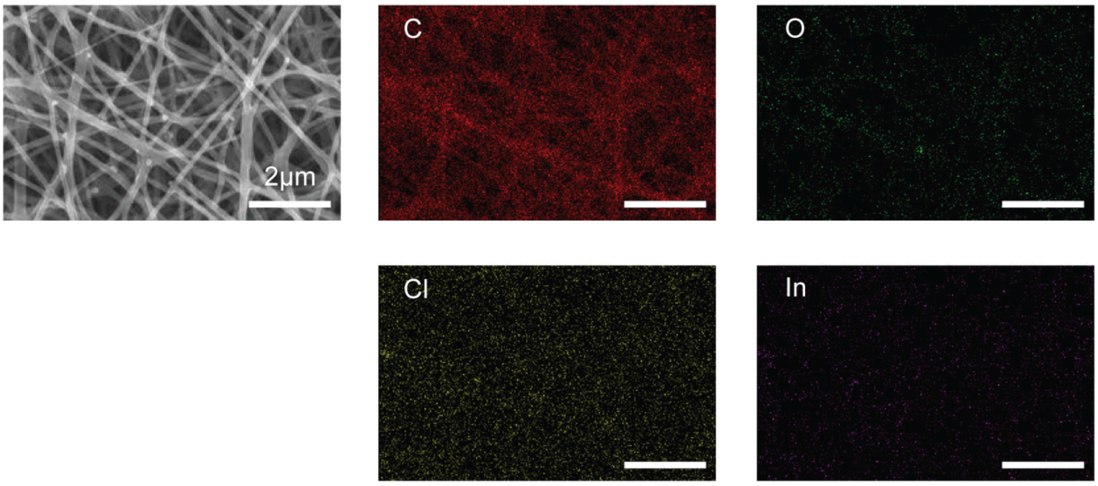


Figure S4. Elemental identifications of the as-prepared CNF with $InCl_3$ under SEM mode. EDS maps of carbon, oxygen, chlorine, and indium, respectively.

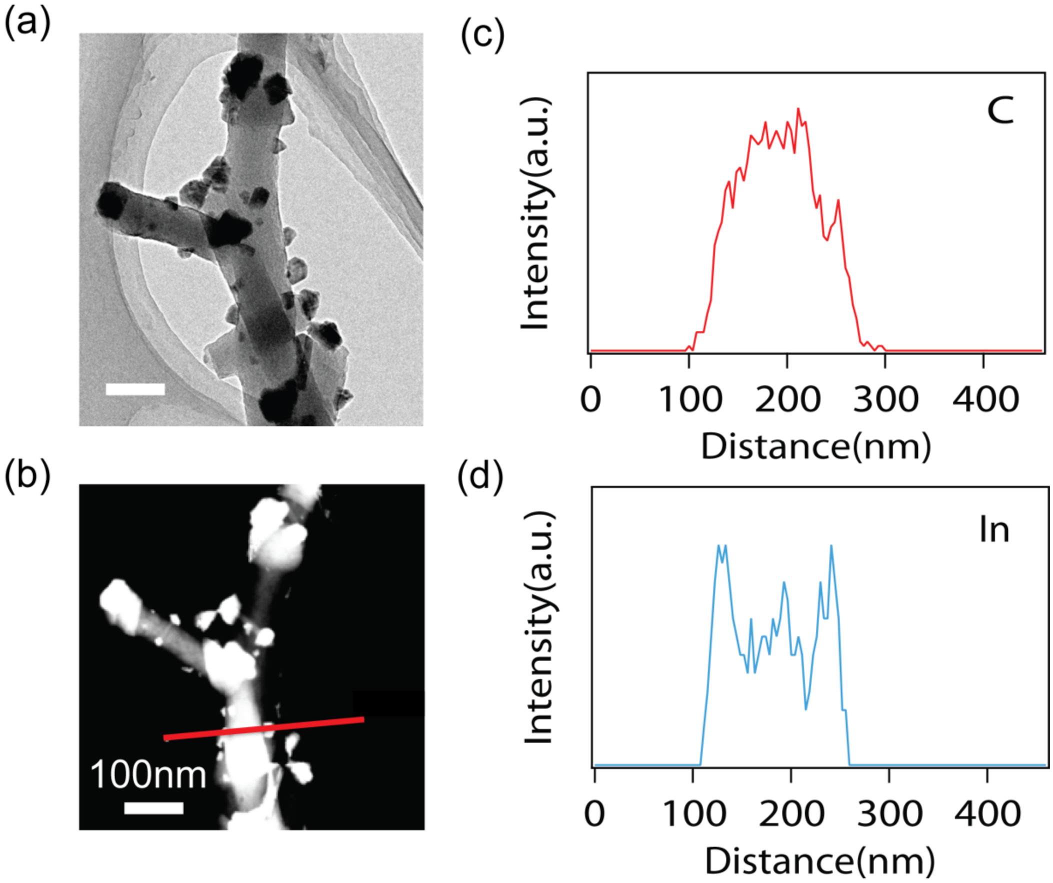


Figure S5. TEM (a) and STEM (b) images of a typical $InCl_3$-C nanofiber. The line scan results show the carbon (c) and indium (d) distributions corresponding to the red line in (b).

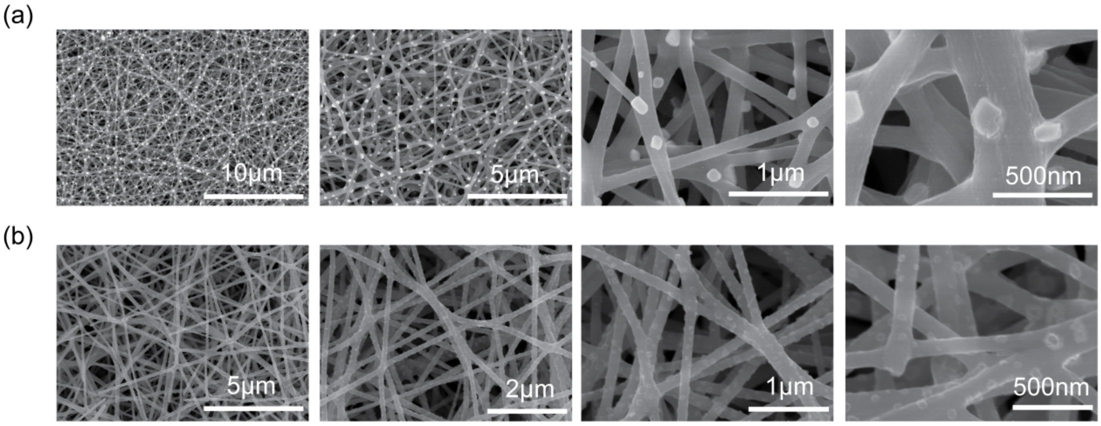


Figure S6. SEM images of CNF with (a) $MgCl_2$ (b) $CaCl_2$ on the surface.

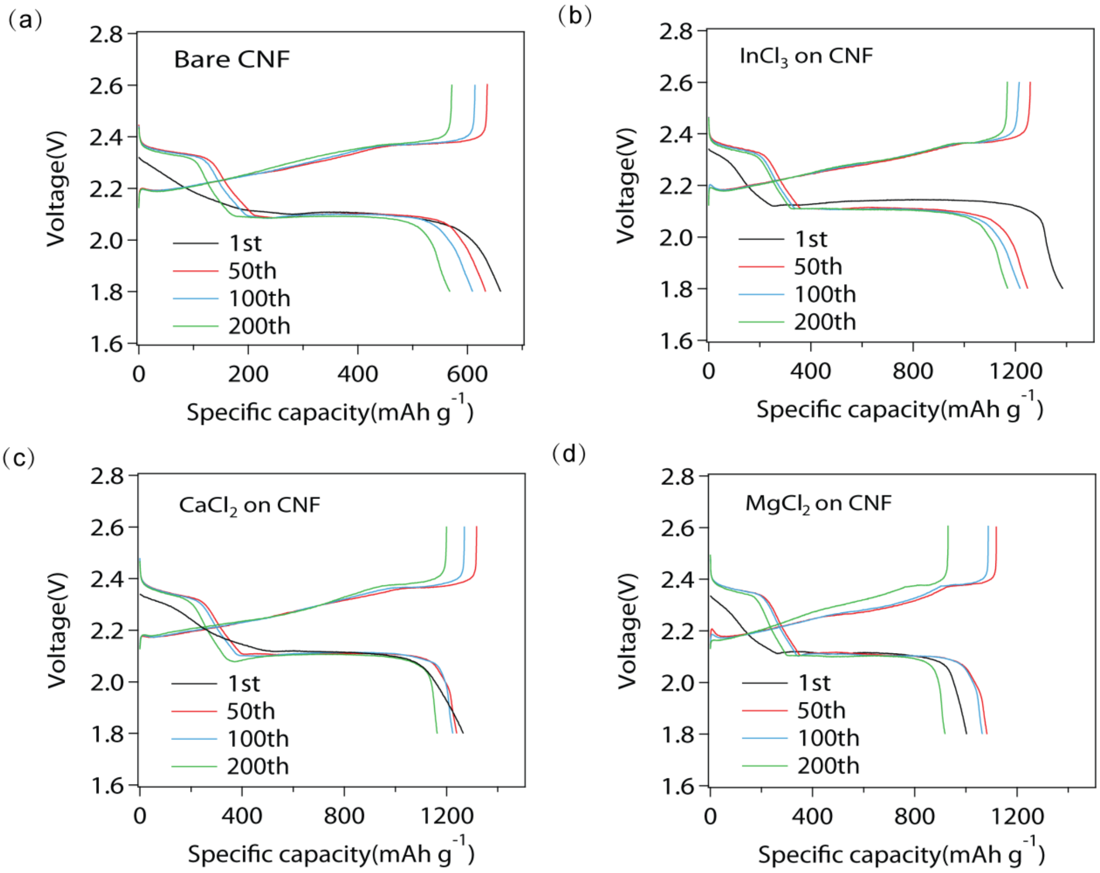


Figure S7. Voltage *vs.* capacity profiles of the first 200 cycles at 0.2C in the voltage range of 1.8-2.6V (*vs.* $Li^+/Li$) for Li-S cells using bare carbon nanofiber (a) and for Li-S cells containing $InCl_3$ (b), $CaCl_2$ (c), or $MgCl_2$ (d).

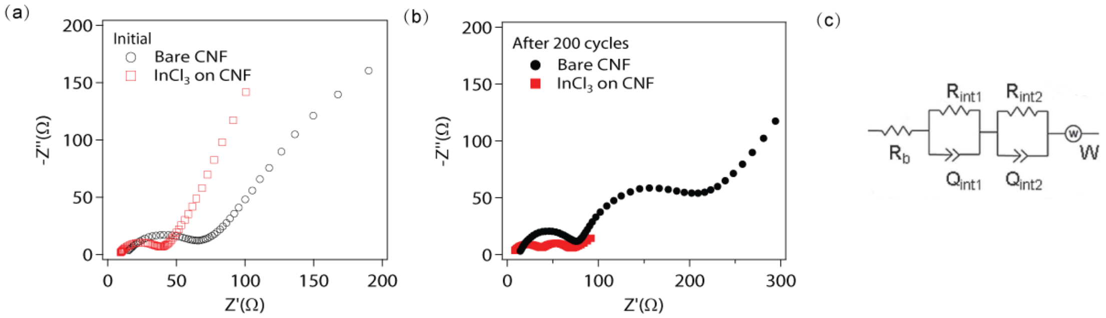


Figure S8. Impedance spectra of Li-S cells containing bare CNF or $InCl_3$-C before (a) and after cycling (b). The equivalent circuit model is provided in (c), where $R_b$ is the bulk resistance, $R_{int1}$ and $R_{int2}$ are the interfacial resistances, $Q_{int1}$ and $Q_{int2}$ are the corresponding constant phase element capacitances and W is the Warburg diffusion element.

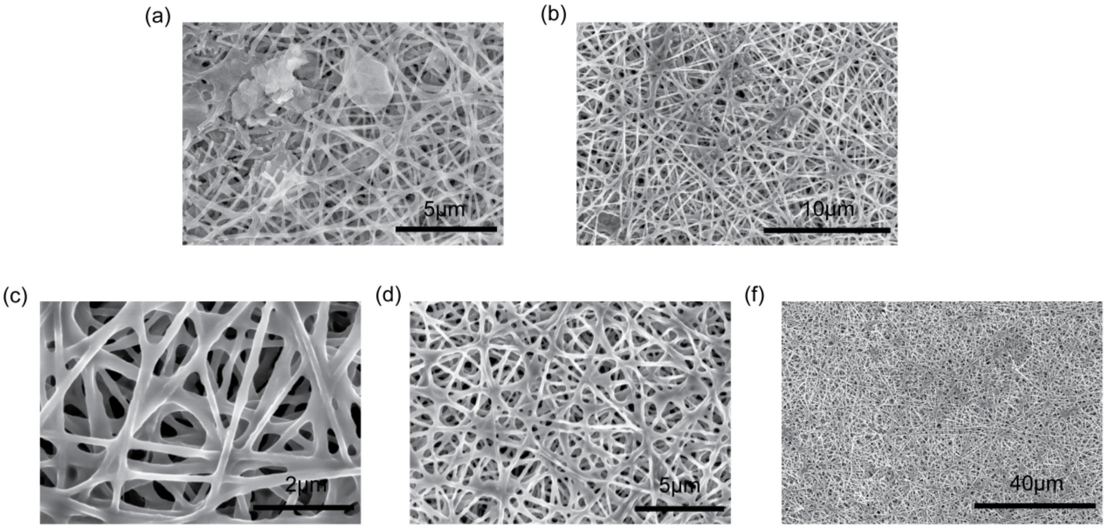


Figure S9. Post-mortem SEM images of cathodes with bare CNF (a)(b) or with $InCl_3$-coated CNF (c)-(f) at different magnifications.

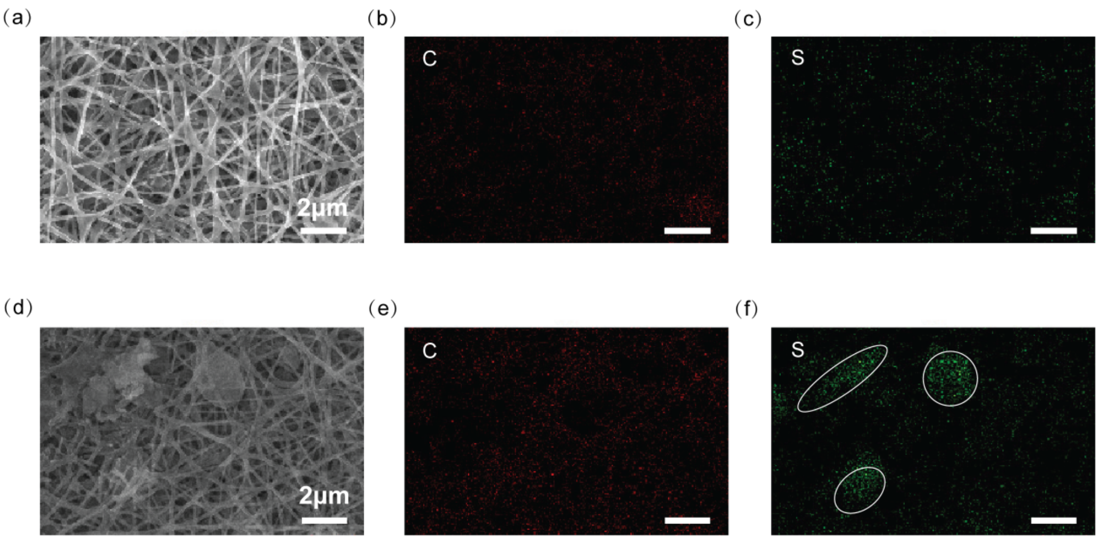


Figure S10. Post-mortem SEM and EDX maps of cathodes with $InCl_3$ (a)-(c) and without $InCl_3$ (d)-(f).

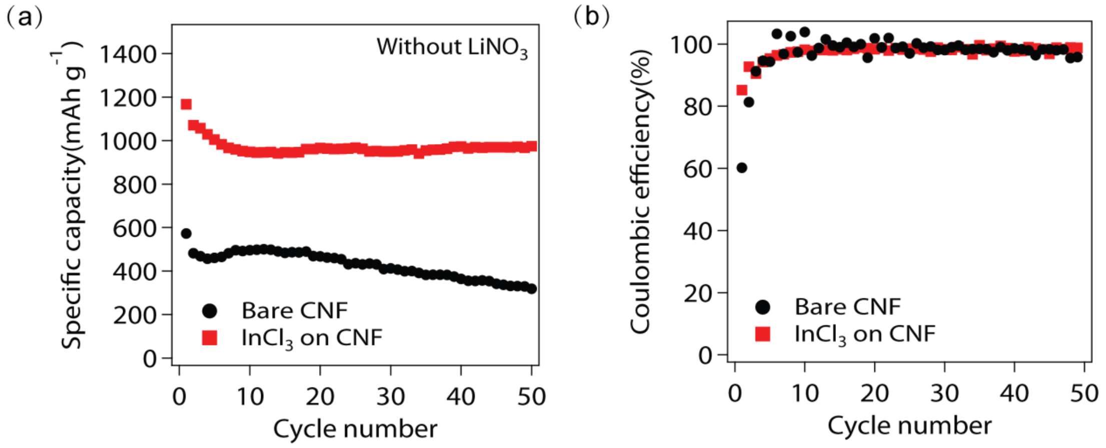


Figure S11. Cycling performance of cells without $LiNO_3$ additives. The galvanostatic measurements were carried out at 0.2C with moderate S loading, 2mg cm$^{-2}$.

## References

[1] G. Kresse, J. Furthmüller, *Phys. Rev. B* **1996,** 54, 11169.

[2] J. P. Perdew, K. Burke, M. Ernzerhof, *Phys. Rev. Lett.* **1996,** 77, 3865.

[3] J. P. Perdew, K. Burke, M. Ernzerhof, *Phys. Rev. Lett.* **1997,** 78, 1396.

[4] G. Kresse, D. Joubert, *Phys. Rev. B* **1999,** 59, 1758.'

[5] N. A. Tumanov, E. Roedern, Z. Lodziana, D. B. Nielsen, T. R. Jensen, A. V. Talyzin, R.

Cerny, D. Chernyshov, V. Dmitriev, T. Palasyuk, and Y. Filinchuk, Chem. Mater. **2016,** 28, 274.

[6] J. Liu, Q. Sun, Y. Kawazoe, and P. Jena. Phys. Chem. Chem. Phys., **2016,** 18, 8777.

[7] L. Ma, S. Wei, H. L. Zhuang, K. E. Hendrickson, R. G. Hennig, L. A. Archer, *J. Mater. Chem. A* **2015,** 3, 19857.

[8] M. Dion, H. Rydberg, E. Schröder, D. C. Langreth, B. I. Lundqvist, *Phys. Rev. Lett.* **2004**, 92, 246401.

[9] K. Lee, E. D. Murray, L. Kong, B. I. Lundqvist, D. C. Langret, *Phys. Rev. B* **2010,** 82, 081101.

[10] Q. Zhang, Y. Wang, Z. W. Seh, Z. Fu, R. Zhang, Y. Cui, *Nano Lett.* **2015**, 15, 3780.
